# Recovery rate data for silicon nitride nanoparticle isolation using sodium polytungstate density gradients

**DOI:** 10.1016/j.dib.2018.06.019

**Published:** 2018-06-19

**Authors:** J. Patel, S. Lal, S.P. Wilshaw, R.M. Hall, J.L. Tipper

**Affiliations:** a Faculty of Biological Sciences, University of Leeds, UK; b School of Mechanical Engineering, University of Leeds, UK

## Abstract

The average recovery rate of silicon nitride nanoparticles isolated from serum using the method detailed in previous article “A novel method for isolation and recovery of ceramic nanoparticles and metal wear debris from serum lubricants at ultra-low wear rate” (Lal et al., 2016) [1] was tested gravimetrically by weighing particles doped into serum before and after the isolation process. An average recovery rate of approximately 89.6% (± 7.1 SD) was achieved.

**Specifications Table**

**Table:** 

Subject area	Biology
More specific subject area	Biomaterials
Type of data	Table
How data was acquired	High-resolution microbalance (Mettler XP26)
Data format	Raw
Experimental factors	Serum samples were doped with 3.44 mg (1 mm^3^) of silicon nitride particles using a microbalance to weigh particles
Experimental features	Samples were subjected to the particle isolation process given in [1] and isolated particles were weighed using a microbalance
Data source location	N/A
Data accessibility	Data is with this article
Related research article	Patel et al. [2]

**Value of the data**

• The recovery rate for silicon nitride nanoparticles was obtained using an alternative method to that given in [1], thus the data from both can be compared for further insight.
• This data may be used to compare the recovery rate for silicon nitride nanoparticles obtained using sodium polytungstate gradients to any future isolation methods for the same particle type, or to establish a benchmark for future method improvement.
• Researchers may wish to use the methods given here to obtain a recovery rate for other particle types, other isolation methods, or for other applications.

## 1 Data

Details of the commercial silicon nitride nanoparticles used in this study, including the results of scanning electron microscopy, elemental analysis and particle characterisation, are given in [1] and [2]. The data presented here are the percentage recovery rates by mass for each of three serum samples doped with silicon nitride particles, in addition to a control serum sample to which no particles were added, following the isolation process outlined in [1]. Each replica particle sample decreased in weight by varying degrees, while the control sample remained a similar weight (Table 1). An average recovery rate of approximately 89.6% (± 7.1 SD) of the silicon nitride particles was achieved (Table 1). Scanning electron microscopy and elemental analysis of the isolated particles verified that the recovered masses consisted of silicon nitride particles with few contaminants.

**Table 1:** Calculation of percentage recovery rates for silicon nitride nanoparticle-doped serum samples.

	Initial particle doping mass (equivalent to 1 mm^3^)	Net weight of recovered particles (g)	Net weight of recovered particles (g) - net weight of control sample	Weight of recovered particles (%)
Replica 1	0.00344	0.003371	0.003365	97.8
Replica 2	0.00344	0.002775	0.002769	80.5
Replica 3	0.00344	0.003116	0.003110	90.4
Control	–	0.000006	–	–
Average				89.6 ± 7.1 SD

## 2 Experimental design, materials, and methods

Silicon nitride nanoparticles (< 50 nm, Sigma-Aldrich, UK) were weighed after 24 h acclimation time in a measurements laboratory using a high-resolution microbalance (Mettler XP26) and added to volumes of 14 mL of 25% (v/v) foetal bovine serum. For the sample masses of 3.44 mg used, the accuracy of the microbalance measurements was better than ±0.03%. Samples were mixed by vortexing and sonicating for 20 min three times and incubated at 37 °C for 16–20 h to allow proteins to bind to particles. Samples were subjected to the isolation process as detailed in [1], which involved serum concentration by ultracentrifugation, enzymatic digestion of serum proteins with proteinase K, density gradient ultracentrifugation using sodium polytungstate density gradients and removal of excess sodium polytungstate by further rounds of ultracentrifugation. Isolated particles were resuspended in sterile filtered water (Baxter, UK) by vortexing and sonicating for 20 min three times and filtered onto 0.015 µm pore-sized pre-weighed filters. The filters were re-weighed to determine the mass of particles recovered. A control sample, containing serum with no added particles that was also subjected to the recovery process, was also filtered and weighed; any increase in weight was deducted from the weight of the recovered particle-containing filters. Recovery as a weight % was calculated. Filtered particles were inspected by scanning electron microscopy and elemental analysis as detailed in [2] to ensure that the recovered mass was not due to contamination.

## References

[1] Lal S., Hall R.M., Tipper J.L. (2016). A novel method for isolation and recovery of ceramic nanoparticles and metal wear debris from serum lubricants at ultra-low wear rates. Acta Biomater..

[2] Patel J., Lal S., Nuss K., Wilshaw S.P., Rechenberg B., Hall R.M., Tipper J.L. (2018). Recovery of low volumes of wear debris from rat stifle joint tissues using a novel particle isolation method. Acta Biomater..

