# Supplementary material for: Recovery rate data for silicon nitride nanoparticle isolation using sodium polytungstate density gradients
Source: Data Brief. 2018 Jun 19;19:1474–6. doi: 10.1016/j.dib.2018.06.019 (PMC6141149; doi:10.1016/j.dib.2018.06.019)
Supplement: Supplementary file 1 — Transparency document [file mmc1.docx]

**Conflicts of interest**

There are no conflicts of interest.
